# Niche breadth specialization impacts ecological and evolutionary adaptation following environmental change

**DOI:** 10.1093/ismejo/wrae183

**Published:** 2024-09-26

**Authors:** Cécile Gubry-Rangin, Axel Aigle, Leonel Herrera-Alsina, Lesley T Lancaster, James I Prosser

**Affiliations:** School of Biological Sciences, University of Aberdeen, 23 St Machar Drive, Aberdeen AB24 3UU, United Kingdom; School of Biological Sciences, University of Aberdeen, 23 St Machar Drive, Aberdeen AB24 3UU, United Kingdom; Present address: Mexbrain, Villeurbanne, France; School of Biological Sciences, University of Aberdeen, 23 St Machar Drive, Aberdeen AB24 3UU, United Kingdom; School of Biological Sciences, University of Aberdeen, 23 St Machar Drive, Aberdeen AB24 3UU, United Kingdom; School of Biological Sciences, University of Aberdeen, 23 St Machar Drive, Aberdeen AB24 3UU, United Kingdom

**Keywords:** community assembly, archaea, generalist, specialist, pH, transition, disturbance, soil

## Abstract

Ecological theory predicts that organismal distribution and abundance depend on the ability to adapt to environmental change. It also predicts that eukaryotic specialists and generalists will dominate in extreme environments or following environmental change, respectively. This theory has attracted little attention in prokaryotes, especially in archaea, which drive major global biogeochemical cycles. We tested this concept in Thaumarchaeota using pH niche breadth as a specialization factor. Responses of archaeal growth and activity to pH disturbance were determined empirically in manipulated, long-term, pH-maintained soil plots. The distribution of specialists and generalists was uneven over the pH range, with specialists being more limited to the extreme range. Nonetheless, adaptation of generalists to environmental change was greater than that of specialists, except for environmental changes leading to more extreme conditions. The balance of generalism and specialism over longer timescales was further investigated across evolutionary history. Specialists and generalists diversified at similar rates, reflecting balanced benefits of each strategy, but a higher transition rate from generalists to specialists than the reverse was demonstrated, suggesting that metabolic specialism is more easily gained than metabolic versatility. This study provides evidence for a crucial ecological concept in prokaryotes, significantly extending our understanding of archaeal adaptation to environmental change.

## Introduction

Ecological theory predicts that the distribution and abundance of organisms depend on their environmental tolerances and associated trade-offs in adapting to environmental change. Attempts to increase understanding of the distribution of organisms have led to their classification as specialists (narrow niche breadth) and generalists (wide niche breadth). The “Jack-of-all-trades, master of none” hypothesis [1] proposes that specialists are predicted to outcompete generalists in more extreme environments, where the costs of generalism may be particularly detrimental. In contrast, generalists are predicted to outperform specialists in changing environments due to their broader tolerances [2]. Evolutionary dynamics can also impact these patterns. Generalism, which often involves a more diverse set of environmental adaptations, is typically considered more difficult to gain evolutionarily than specialism. Alternatively, specialist lineages may be more easily lost, as restriction to narrow environmental subsets can limit lineage diversification opportunities. Thus, the maintenance and distributions of generalists and specialists reflect balance among competing processes of adaptability, diversification and environmental variation and extremes, over short- and long-term timescales.

These concepts of niche specialization have attracted much attention in eukaryotes [3], explaining species’ spatial- and trait-specialization and answering fundamental questions linked to fitness advantage, rates of speciation and resistance to extinction. Testing the importance of niche specialization for prokaryote distributions is much less common, despite a few exceptions for several well-studied cultivated microorganisms (mainly bacteria such as *Escherichia coli* and *Pseudomonas fluorescens*) used in long-term experimental evolution studies [4] or competition experiments [5]. However, studies in these non- or weakly-diversified microbial populations did not consider the unstable conditions that a natural environment provides. In parallel, patterns of ecological specialization and associated abundance-specificity relationships have been analyzed in natural complex microbial populations, focusing on spatial distributions through occurrence in single or multiple locations (e.g. [6–11]) or environmental trait-based niche breadth (e.g. [12–16]). However, to our knowledge, the ecological success of microbial specialists and generalists in natural environments and how these respond under the influence of environmental disturbance have rarely been explored [17] and never attempted in terrestrial environments. Analysis of evolutionary mechanisms involved in microbial niche specialization has only been rarely investigated, often revealing that the transition of widespread soil bacteria from generalist to specialist state was more frequent than the reverse [10, 11, 18]. Therefore, this study aimed to determine the generality of the ecological and evolutionary theory of niche specialization by testing predictions of changes in prokaryotic communities along gradients of environmental extremes and following abrupt environmental change. Testing this theory in prokaryotes is essential, as they are highly abundant organisms with major roles in biogeochemical cycles in all environments. It is also important for predicting microbial community composition and activity following environmental changes and for understanding maintenance of the high microbial diversity on Earth.

Our chosen model microorganisms are ammonia oxidizing archaea (AOA), belonging to the Nitrososphaeria class, which are abundant prokaryotes with high physiological and metabolic versatility [19, 20], ubiquitous over an extensive range of soil pH [21] with a key impact on ecosystem function [22]. As for many microbes over a large range of phylogenetic scales [23, 24], pH is a major abiotic factor associated with their distribution and abundance in soil [21]. Their evolutionary diversification has also been associated with changes in pH preferences over deep evolutionary history [25, 26]. Therefore, soil pH provides an important trait-based factor for investigating ecological and evolutionary niche specialization.

Here, the adaptation of specialists and generalists (defined based on their niche breadth index) was assessed in both ecological and evolutionary dimensions to understand their relative dominance across environments and following an environmental disturbance. This assessment also aimed to determine whether specialism or generalism exhibits greater evolutionary conservatism in microbes and how these strategies differentially contribute to diversification dynamics. In this context, the following hypotheses were tested: (i) specialists and generalists exist across the range of soil pH, with common dominance of generalists, due to high metabolic versatility, but dominance of specialists toward the limits of the pH range, due to increasing benefits of metabolic specialization and increasing costs of generalism; (ii) generalists have greater fitness than specialists following environmental pH disturbance, due to higher metabolic versatility, except for transitions to extreme pH, where specialists outcompete generalists; (iii) generalists have higher speciation rates than specialists, due to their high gene acquisition enabled by survival in an extensive range of environments, thereby increasing diversification rate, whereas the restricted niche of specialists decreases gene gain and enhances extinction rate; (iv) the rate of transformation from generalists to specialists (i.e. transition rate) is greater than the reverse across the evolutionary history of Thaumarchaeota, as genome streamlining and associated selective pressures can facilitate specialization of widespread generalists to specific environments, whereas acquisition of multiple functional distinct gene sets in specialists is less likely. Hypotheses 1 and 2 were evaluated over short timescales through changes in activity, reflecting physiological responses, and long timescales by the presence or absence of selected microbial members, reflecting processes of establishment and extinction. Testing niche specialization theory in prokaryotes in natural communities provides a unique opportunity to analyze the key contributors of adaptation in the ecosystem, as the distinction between the resident cells (representing all cells present in a particular environment) and active (currently adaptive) cells cannot often be incorporated into studies on eukaryotes (but see resurrection ecology studies [27]). Hypotheses 3 and 4 focused on the deep evolutionary timescale (i.e. over 950 million years), where diversification and evolutionary transitions were likely influential [25, 28].

## Material and methods

### Construction of a specialist/generalist database

A previously published database of a 629-bp ammonia monooxygenase subunit A *amoA* AOA was used in this study. This database used 454-sequencing of the *amoA* gene performed on 47 soils from the United Kingdom with soil pH ranging from 3.48 to 8.74 [21, 26]. This database represents the worldwide distribution of AOA with regards to pH phylogenetic distribution [21], encompassing all known terrestrial clades. As most AOA have a single *amoA* gene copy in their genome (apart from two out of 135 genomes [19]), it is assumed that each *amoA* sequence was derived from an individual archaeal cell. A series of strict quality filtering steps were performed before assembling forward and reverse sequences (see details in [21]). The resulting 108 192 *amoA* sequences were distributed into 18 858 amplicon sequence variants (ASVs) at 100% sequence identity. Following removal of singletons and other spurious contigs, all ASVs were dereplicated at 97.5% sequence identity using Uclust [29], resulting in 425 phylotypes [21]. This phylotype assignment provided relative abundance matrices for the 47 soils. These matrices were subsequently transformed into matrices of AOA total cell abundance (estimated by specific quantitative polymerase chain reaction [qPCR]) [21].

pH niche specialization was estimated using hydrogen ion concentration rather than pH, avoiding the distortive effect of this logarithmic trait, but we refer to niche specialization as “pH niche specialisation” hereafter rather than “[H^+^] niche specialisation” for simplicity. The pH niche breadth of each phylotype is related to the range of soil pH plots in which it was found. The pH niche breadth distribution of all phylotypes present in more than one soil was used to classify them into generalist and specialist states, based on a modified Levin’s niche breadth index [30]. This index (*B*) is based on the distribution of phylotypes among soils of different pH using the following formula:


$$ B_j=\frac{1}{\sqrt{n_j}}\cdotp \frac{{\sigma _j}^2}{{\mu_ j}^2} $$

where *B_j_*, *σ_j_* and *μ_j_* indicate the habitat niche breadth, pH standard deviation and pH mean of phylotype *j*, respectively, whereas *n_j_* indicates the number of samples in which the phylotype *j* is observed. This index was selected as the standard deviation (*σ_j_*) and the standard error (*σ_j_ / √n_j_)* were both individually skewed toward each side of the abundance gradient, whereas the selected B index showed a homogeneous distribution of the niche breadth index (Supplementary Fig. S1). Homogeneous distribution of the index across the abundance gradient is desirable to minimize bias in the attribution of the specialist/generalist trait. Although niche breadth was attributed to soil pH, the influence of other confounding factors (e.g. soil characteristics, vegetation types) cannot be excluded. Nonetheless, niche breadths attributed to natural variation in pH corresponded to expected responses under experimental pH regimes in the laboratory, see below.

The median of the niche breadth values was chosen as the arbitrary cut-off to discriminate between specialist (lower than the median) and generalist (higher than the median) categories. The niche breadth of the remaining phylotypes was null due to their presence in a single soil, and they were classified as putative-specialists hereafter.

### Soil incubations

Two sets of soil incubation were performed: a native soil experiment and an environmental change experiment. Some approaches and measures of AOA growth and activity were published in a previous study [31]. Here, we analyzed the AOA community sequencing and associated niche breadth, which were not described previously.

For the native soil experiment, three pH plot soils were sampled on 6 June 2015 from the upper 10 cm of plots of a sandy loam agricultural soil pH gradient (SRUC, Craibstone, Scotland; grid reference NJ872104) maintained at pH values of approximately 4.5, 6, and 7.5 since 1961 using lime and ferric sulfate, with pH values of 4.77, 6.26, and 7.39 at the time of sampling [31]. Each soil was incubated in microcosms in the dark at 25°C for 30 days under constant moisture content (30%) and aerobic conditions. For the pH disturbance experiment, a single soil, the pH 6.0 soil, was either maintained at pH 6.0 or adjusted to pH 4.5 or 7.5 using lime and ferric sulfate and subsequently incubated in microcosms for 30 days at 25°C (see details in [31]). Osmotic pressure and other related parameters were similar in the different soils.

Two sets of parallel, triplicate microcosms were established for each experiment for each soil condition. Microcosms consisted of 50 g soil to 250 ml Duran bottles and a sub-sample was taken after incubation for 0, 1, 3, 10, and 30 days. Deoxyribonucleic acid (DNA) and ribonucleic acid (RNA) were extracted separately from 0.5 g soil at each time point, and complementary DNA (cDNA) was generated from purified RNA [31]. Stable isotope probing (SIP) was performed in a separate set of microcosms consisting of sealed 120-ml serum bottles with a headspace containing either ^13^C–CO_2_ or ^12^C–CO_2_ (5% (v/v)). DNA was extracted after incubation for 30 days and ultracentrifugation of DNA from ^12^C- and ^13^C–CO_2_ incubations in CsCl gradients was performed (see details [31]). qPCR was used to quantify AOA *amoA* gene abundance in cDNA, DNA, and SIP-DNA (see details in [31]).

### AOA community sequencing in soil incubation experiments

The *amoA* gene sequencing was performed for the resident AOA at each time point using the DNA and cDNA approaches. These data were then used to derive estimates of AOA growth and transcriptional activity (see below). In addition, *amoA* gene sequencing was performed for the active AOA detected by the SIP approach. For the DNA-SIP analyses, ^13^C assimilation was measured as the abundance of *amoA* genes in the ^13^C–DNA fractions in which *amoA* abundance was higher in ^13^C- than in ^12^C-microcosms. Three to six selected ^13^C–DNA fractions representative of the ^13^C-DNA peak were pooled for subsequent sequencing for each sample.

Archaeal *amoA* genes were amplified using primers CrenamoA23f/CrenamoA616r containing additional specific MiSeq-tailed sequences, following manufacturers’ recommendations, and following the protocol described previously [32]. For each sample, all amplifications were performed in triplicate 25-μl reactions using the KAPA HiFi HotStart ReadyMix (Kapa Biosystems) with 0.4 μM of each primer and 40–60 ng of template. Thermal cycling conditions were 95°C for 3 min, followed by 35 cycles of 98°C for 20 s, 58°C for 15 s, 72°C for 20 s, followed by 72°C for 5 min. Following individual quality assessment, triplicate reactions were pooled, PCR-amplified sequences were cleaned using AMPure® XP beads (Beckman Coulter), and PCR-indexing was performed using the Nextera XT Index Kit according to the manufacturer’s protocol. Following further cleaning, library quantification and normalization, paired-end V3 MiSeq sequencing (Illumina) was performed, producing 2 × 300-bp reads. As the sequencing was conducted as part of a large sequencing project (unpublished data), samples were run in multiple runs provided by several companies (Macrogen, NCIMB, and NBAF).

Due to the length of the *amoA* fragment (629-bp without adapters) and the size limitation of the paired-end V3 MiSeq sequencing approach, we applied an in-house read joining strategy [32]. In short, after read demultiplexing, paired-end raw reads were trimmed and filtered using Trim Galore V0.4.5 [33] and DADA2 [34], ensuring maintenance of the coding frame. The reverse reads were reverse complemented and concatenated with the forward reads. The assembled reads were then dereplicated at 100% sequence identity using usearch [29]. Finally, dereplicated concatenations were translated, and any sequence that included a stop codon was deleted before removing chimeras and singletons using unoise3 [35]. These reads were then compared to the pH niche breadth database using Blastn (−max_target_seqs 1) [36] to assess their phylotype identity in the database. AOA taxonomy was inferred based on a previously published *amoA* database [37].

### Presence, growth, and activity measurements of each phylotype in the soil experiments

The abundance of each phylotype was estimated for each soil and time point combination using their relative abundance (from the *amoA* sequencing approach) and the sample qPCR estimates. Growth, transcriptional activity, and DNA replication of each phylotype were assessed by analysis of temporal changes in *amoA* gene abundance, *amoA* transcript abundance and incorporation of ^13^C-CO_2_ into *amoA* genes, respectively. Therefore, the abundance of each active phylotype corresponded to its differential abundance between the final and initial time points (for its *amoA* gene abundance) or between successive sample time points (for *amoA* transcript abundance). In both cases, if the differential abundance was negative, abundance was reported as null. For each phenotype (specialist, generalist, or putative-specialist), phylotype abundances were summed within each soil to assess the respective fitness of specialists and generalists, as ecosystem function depends on the organismal functional capacity and the number of organisms performing a specific function. In the soil experiments, we therefore refer to five datasets hereafter. Two datasets refer to the resident AOA (i.e. total or present, including active, dormant, and dead or remnant), one being the “DNA-resident” or “amoA gene abundance” (DNA-based abundance at a specific time point) and the other being the “RNA-resident” or “amoA transcript abundance” (RNA-based abundance at a specific time point). The other three datasets refer to the active AOA, including the “DNA-active” or “growth” (DNA-based temporal change in abundance), “RNA-active” or “transcriptional activity” (RNA-based temporal change in abundance) and the “SIP-active” or “DNA replication” (SIP-based labeling incorporation).

### Distribution of the two phenotypic states along an environmental gradient and in the incubated soils

The proportions of specialists and generalists were initially compared over the pH range in the soil database (pH 3.48 to 8.74) using a best-fitting non-linear regression model. The 47 soils were also grouped into two categories: (i) “classical” pH range soils (5 < pH < 8.5) or (ii) extreme range pH soils (pH ≤ 5). Proportions of specialists or generalists were then compared in the two categories. For all statistical tests comparing proportions or abundances between two groups, a Welch two-sample *t*-test was performed if data were normally distributed (tested with a Shapiro–Wilk test) and residuals had homogeneous variances (tested with a Bartlett test). A Kruskal–Wallis test was performed if these assumptions were not met. All analyses were performed in R v4.0.3 [38] using the vegan v2.5–6 [39], ggplot2 v3.3.2 [40], nlme v3.1 [41], and sjPlot v2.8 [42] packages.

The abundances of specialists and generalists were also compared within each soil for the native and pH-perturbed incubated soils. The resident AOA community was analyzed in both experiments and the active AOA community was examined for the environmental change experiment. The proportion of specialists or generalists was compared in the intermediate and the lowest or highest pH soils for these two experiments, and the abundance of the total active cells was also compared between the three soils in the environmental change experiment.

### Microbial community structure, diversity, and dormancy

Phylogenetic signal of the generalism trait was tested using the phylosignal function in the package picante v1.8.2 [43] using the previously published maximum-likelihood phylogenetic tree containing 370 phylotypes (following detection of recombination events in the remaining 55 phylotypes) [25] and the specialist/generalist database.

Beta diversity was estimated for each DNA or cDNA dataset using the vegdist function with default parameters in conjunction with the Bray–Curtis distance metric. Ordination plots were performed by nonmetric multi-dimensional scaling using the function metaMDS. Differences in the Bray–Curtis distance metrics over soil pH or time were analyzed with permutational multivariate analysis of variance using the analysis of similarity function with 999 permutations.

Shannon diversity index was calculated using the number and abundance of each phylotype for the total resident and active AOA communities within the three pH-perturbed incubated soils after incubation for 30 days. Both alpha- and beta-diversity indexes were estimated without prior rarefaction [44]. The proportion of combined dormancy and death (corresponding to the inactive community) was estimated in the same incubated soils as the complement of the ratio of numbers of active to resident cells. Given the high variability of RNA data, statistical analyses of the Shannon diversity index and combined dormancy and death were restricted to the DNA and SIP approaches. Changes in Shannon diversity in the different soils were analyzed using linear modeling with the type (resident or active), nature (DNA, RNA, SIP), and pH (pH 4.5, pH 6.0 and pH 7.5) as fixed factors. A second linear model was applied with the type (DNA-resident, DNA-active, SIP-active), and pH change (native, perturbed) as fixed factors. Changes in combined dormancy and death in the different soils were analyzed using linear modeling with the nature (DNA, SIP) and pH change (native, perturbed) as fixed factors. Analysis of variance and Tukey honestly significant difference tests were performed to assess the significance of differences between soils.

Phylogenetic community structure was measured for the 47 soil samples using mean pairwise distance (mpd) weighted by phylotype abundance and assessing their statistical significance by comparing the empirical mpd against mpd from 999 simulated/null communities, using the package Picante v1.8.2 [43]. Those simulated null communities had the same number of phylotypes as in empirical communities (i.e. “richness” algorithm). The standardized effect size for the mpd metric (ses.mpd) was used to describe relatedness patterns across samples. Three linear models were fitted and compared using ses.mpd for each community as a response variable. The explanatory variable was pH in Model 1 and the relative abundance of specialist phylotypes in the community in Model 2. In Model 3, ses.mpd was a function of both pH and proportion of specialists (and their interaction).

### Niche breadth-dependent diversification

Changes in the value or state of macro-evolutionary-relevant traits will influence diversification rates by increasing or reducing the chances of speciation and extinction events. The state-of-the-art approach involves fitting and comparing models assuming the association or independence of the trait in question and heterogeneity in diversification rates across the phylogenetic tree [45].

The influence of the evolution of pH specialization on macroevolutionary processes was analyzed alongside the influence of geographic extent (range size). Phylotypes recorded in up to three sites were classified as endemic (or geographically restricted), whereas phylotypes present in more sites were classified as cosmopolitan (or geographically widespread). Regarding pH specialization, the putative-specialist phylotypes were considered pH-specialist phylotypes to provide just two pH preference categories: generalists and specialists. Then, the two states of both traits (which yields to a four-state system) were combined so that each phylotype was assigned to one of the following states: endemic-pH specialist, cosmopolitan-pH generalist, cosmopolitan-pH specialist, and endemic-pH generalist.

The joint evolution of pH specialization and geography was modeled as the shift undertaken by a lineage from one of the four states to another. Six evolutionary models were constructed that make different assumptions on which transitions are allowed and how transition rates relate to one another (Supplementary Fig. S2). Model 1, for instance, assumes that the transition rate from pH specialist to generalist state equals that in the opposite direction. However, this rate differs from the rate characterizing range expansion/contraction, i.e. change from endemic to cosmopolitan state (and the reverse). Model 1 (unlike models 2 and 6) assumes no transition is possible between endemic-pH specialists and cosmopolitan-pH generalists.

In combination with the six models of pH and range size evolution, niche-dependent and niche-independent models were used that assumed whether or not lineages systematically experience changes in diversification rates linked to transition events in the system. Additionally, homogeneous rate models were explored in which all lineages diversify at the same rate. The 18 models were fitted using the R package SecSSE v2.6.0 [46], and their likelihoods were compared while considering differences in the number of free parameters (AIC, Akaike Information Criterion) to find the model with the highest statistical support. For each model, three likelihood optimizations were run starting in different parameter space points to prevent finding only local optima.

## Results

### Construction of an ecological niche specialization database

**Figure 1:**
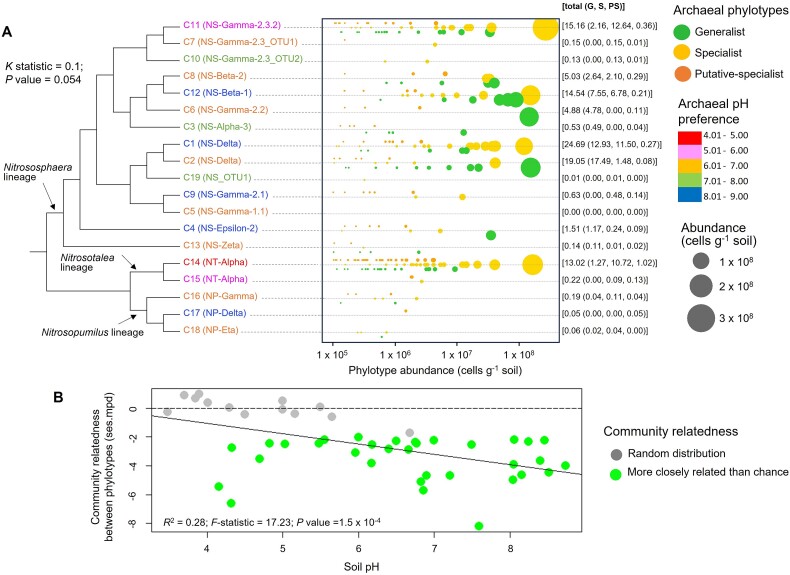
AOA pH niche breadth database and phylogenetic relatedness in 47 UK soil microbial communities distributed across a gradient of pH. (A) Distribution and abundance of archaeal phylotypes across the ammonia monooxygenase (*amoA*) phylogenetic tree. Estimation of the niche breadth index allowed the classification of each phylotype as a generalist (G), specialist (S), or putative-specialist (PS) phylotype. The size of the circles is proportional to phylotype abundance in the database, and the colors of the phylogenetic cluster names correspond to their pH preference [25, 26], as indicated in the legend. There was no evidence for phylogenetic signal or conservatism of the specialist/generalist traits (*K* statistic = 0.1; *P* value = .054). The numbers in brackets at the right of the figure represent the proportion of cells affiliated to each category. (B) The ses.mpd was analyzed against the soil pH. The green circles indicate that phylotypes within this microbial community (present in a single soil) are more closely related than expected by chance (themselves represented as gray circles). The dotted line indicates the neutral model whereas the plain line represents the correlation of evolutionary relatedness of microbial communities with pH level (relationship ses.mpd – pH: *R*^2^ = 0.28; *F* statistic = 17.2; *P* value = 1.5 10^−4^).

The *amoA* sequencing of 47 UK soils distributed across a broad soil pH gradient (pH 3.5 to 8.7) enabled classification of the phylotypes into three phenotypic (trait-specialization) states, pH-generalist (“generalist”, large pH niche breadth), pH-specialist (“specialist”, narrow pH niche breadth), and putative pH-specialist (“putative specialist”, present in a single soil, hence being too rare to classify them as pH-specialists with confidence). This pH niche breadth database encompasses 425 phylotypes and 3.02 × 10^9^  *amoA* sequences; it includes 91 specialists, 87 generalists, and 247 putative specialists, the last representing a low proportion of total sequence abundance (3%; Fig. 1A; Supplementary Table S1). The 425 phylotypes are spread across 19 pH-specialized phylogenetic clusters previously delineated [21, 25], all belonging to the Nitrososphaerales or Nitrosopumilales orders [19].

### Uneven distribution of the two phenotypic states across an environmental gradient

**Figure 2:**
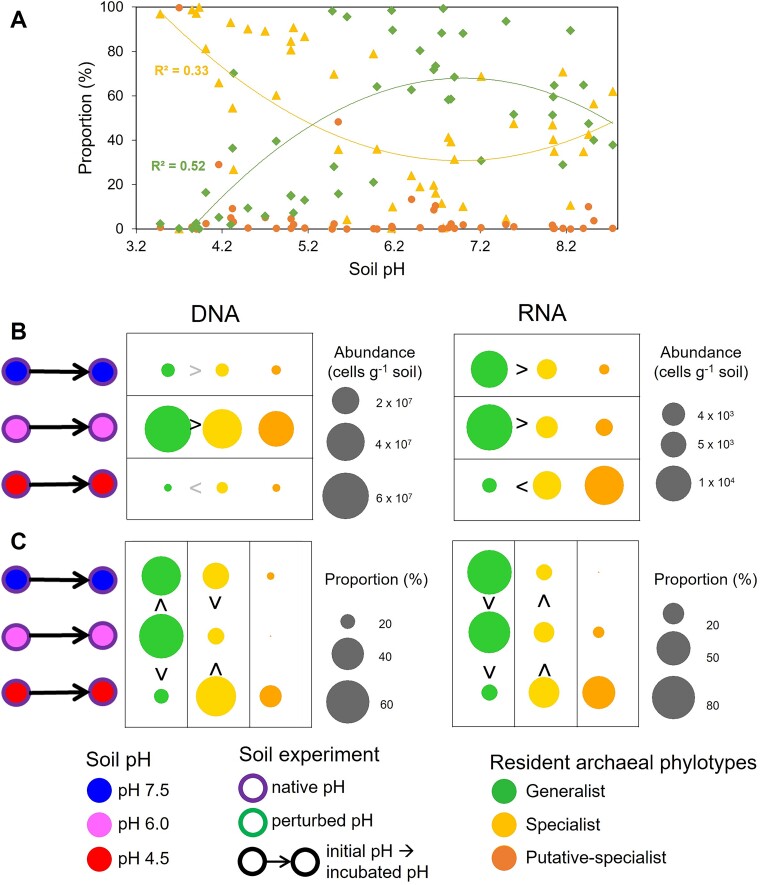
Niche specialization of resident specialists and generalists in native (unperturbed) soils. (A) The proportions of generalist, specialist and putative-specialist resident AOA cells were estimated within 47 non-incubated UK soils, representing the AOA global soil diversity. A non-linear regression model was fitted for the generalist and specialist proportions distribution across the pH range, and the associated correlation coefficients (*R*^2^) are indicated. (B and C) The abundances (B) and proportions (C) of specialist, generalist or putative-specialist AOA cells were estimated within three native pH soils, maintained for 70 years at pH 4.5, pH 6.0, and pH 7.5, after incubation for 30 days at 25°C. The abundances of specialists and generalists were compared within each soil (comparison per line), whereas the proportions of generalists (or specialists) were compared across the soils within each phenotype (comparison per column). Putative specialists were not incorporated in those comparisons. The signs > or < in black and gray represent statistical differences at *P* < .05 and *P* < .1, respectively. The size of the circles is proportional to the abundance or proportion legend associated with each graph.

The selective advantage of generalists over specialists over the pH range was initially tested by comparing the phenotypic state across soil pH within the niche breadth database. The two phenotypic states were distributed across the pH range (Fig. 2A), but polynomial (best fitting non-linear) regression curves indicated that generalists and specialists were generally favored in neutral (*R*^2^ = 0.52) and acidic (*R*^2^ = 0.33) soils, respectively (Fig. 2A; Supplementary Fig. S3). As the pH of most soils on Earth ranges from 3.5 to 10 (https://www.qld.gov.au/), we considered soils with pH ≤ 5.0 and ≥ 8.5 to be approaching the limits of the pH range. As predicted in the first hypothesis, the proportion of generalists was greater than that of specialists, except near the extreme range (pH ≤ 5.0).

Longer-term responses of soil AOA to pH disturbance were investigated by assessing the distribution of resident specialists and generalists alongside a restricted but well-established (70-year) pH-maintained soil plot gradient, following incubation of three soils for 30 days at their native pH (pH 4.5, pH 6.0, or pH 7.5). Despite presence of both specialists and generalists in all soils, generalists were again favored over specialists in non-extreme pH soils (pH 6.0 and pH 7.5), whereas the reverse was true toward the limit range (pH 4.5; Fig. 2B and C). Similarly, the proportion of generalists was lower in the more extreme soil than in the two non-extreme soils.

### Greater fitness of generalists than specialists following an environmental change

**Figure 3:**
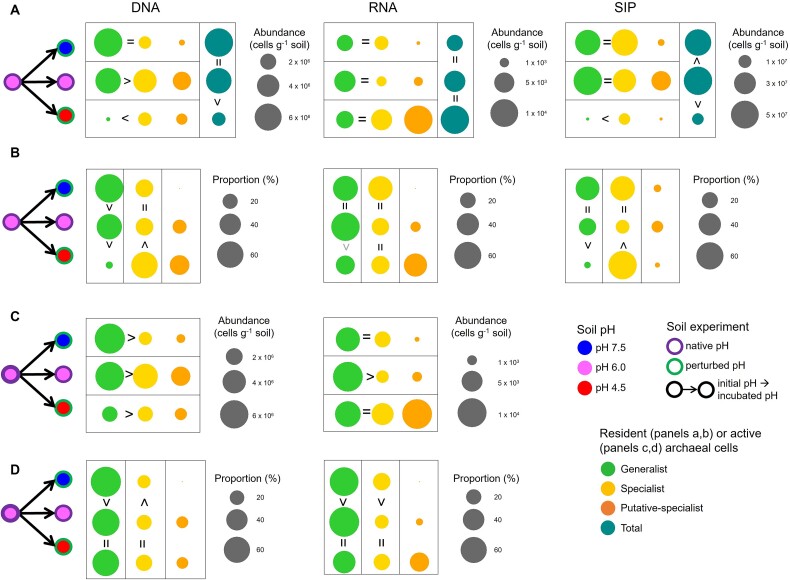
Abundance and proportion of the active and resident specialist and generalist AOA in three incubated pH soils following environmental changes. The abundance (panels A and C) and proportion (panels B and D) of specialist, generalist or putative-specialist AOA cells were estimated within soil microcosms either maintained at their native soil pH (pH 6.0) or subjected to a pH change (from pH 6.0 to pH 4.5 or pH 7.5) for 30 days of incubation at 25°C. For the active cells (panels A and B), their activity was estimated by their growth (DNA), their transcriptional activity (RNA) and their DNA replication (SIP), whereas the presence of resident cells (panels C and D) was estimated by the abundance of gene (DNA) or transcript (RNA). The abundance of specialists and generalists were compared within each soil (comparison per line), and the proportion of generalists (or specialists) was compared across the soils within each phenotype (comparison per column). Putative specialists were not incorporated in those comparisons. The signs > or < in black and gray represent statistical differences at *P* < .05 and *P* < .1, respectively, whereas the symbol = represents no statistical differences between groups. The size of the circles is proportional to the abundance or proportion legend associated with each graph. Data represent the mean and standard errors.

The differential fitness of specialists and generalists following a soil pH change was investigated at the short timescale by incubating soil microcosms for 30 days following an experimental increase or decrease in soil pH, estimating AOA growth and activity during incubation under the native (pH 6.0) or the perturbed (pH 4.5 and pH 7.5) pH conditions. Here, resident (i.e. total present) and active communities were compared, given the potentially high abundance and diversity of dormant or inactive microbial cells in soil [47]. The soil pH change (from pH 6.0 to pH 4.5 or 7.5) induced significant changes in the resident AOA community composition after incubation for 30 days based on both gene and transcript analyses (Supplementary Fig. S4A and B). The AOA community shift happened progressively during incubation for 30 days (Supplementary Fig. S4C–E), suggesting differential AOA growth, dormancy, or death over time. Active AOA were detected in all soils (Fig. 3A; Supplementary Fig. S5A), and the estimated global activity depended on the soil pH and the technique used (with a three-order magnitude difference in AOA activities). Consistent with acidic soils representing extreme environments, AOA growth and DNA replication were lower in the pH 4.5 soil than in the other two soils, whereas this was not the case for the transcriptional activity (see also [31]).

The second hypothesis of a greater fitness of generalists than specialists, except at the extreme range, was tested here by considering only the pH 4.5 soil to be in the extreme range. Indeed, the metabolic requirements for microbial activity are often considered more similar between pH 7.5 and pH 6.0 than between pH 4.5 and pH 6.0, as specific mechanisms such as maintenance of pH homeostasis are required under acidic conditions. Our results suggest greater adaptation of specialists in pH 4.5 than in pH 6.0 and pH 7.5 soils. Analysis of growth and DNA replication generally supported the hypothesis (Fig. 3A and B). The activity of specialists was greater than that of generalists in the pH 4.5 soil based on the AOA growth and DNA replication approaches (Fig. 3A). Generalists were more active than specialists only in the pH 6.0 soil using the AOA growth approach, whereas high variability between replicates may have prevented detection of significant differential growth between generalists and specialists at pH 7.5 (Supplementary Fig. S5B). In addition, the proportion of active generalists was higher in pH 6.0 and pH 7.5 soils than in pH 4.5 soil, while specialists had a fitness advantage in the lower pH soil, as indicated by their higher growth and replication in this soil (Fig. 3B). The transcriptional activity of specialists and generalists did not differ between the different soils (Fig. 3A), which may also be due to high variability in the transcript data (Supplementary Fig. S5B), which would have prevented the detection of differences similar in magnitude to those observed with the other approaches. Importantly, the niche specialization hypothesis was not confirmed when resident cells, rather than active cells, were considered (Fig. 3C and D), demonstrating that the ecological fitness of active and resident microbes to an environmental change was drastically different.

### Impact of environmental change on maintenance of microbial diversity

**Figure 4:**
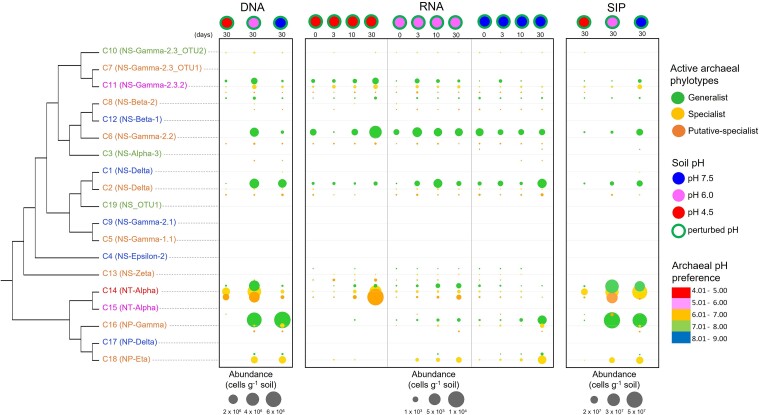
Fine-scale niche adaptation of active specialist and generalist Thaumarchaeota in three incubated pH soils following environmental changes using three activity estimations. The community composition of active communities strongly depended on the soil pH disturbance. The abundance and phylogenetic affiliation (to the 19 phylogenetic clusters previously published [20]) of the active specialist, generalist or putative-specialist AOA cells were estimated within soil microcosms either maintained at their native soil pH (pH 6.0) or subjected to a pH change (from pH 6.0 to pH 4.5 or to pH 7.5) and incubated for 30 days of incubation at 25°C. The activity of AOA cells was estimated by their growth (DNA) and DNA replication (SIP) after incubation for 30 days and by their transcriptional activity (RNA) at four time points (after 1, 3, 10, and 30 days, represented from left to right within each soil). The number of days post-incubation is indicated below the top soil circle. The size of the circles is proportional to the abundance legend associated with each activity estimation.

**Figure 5:**
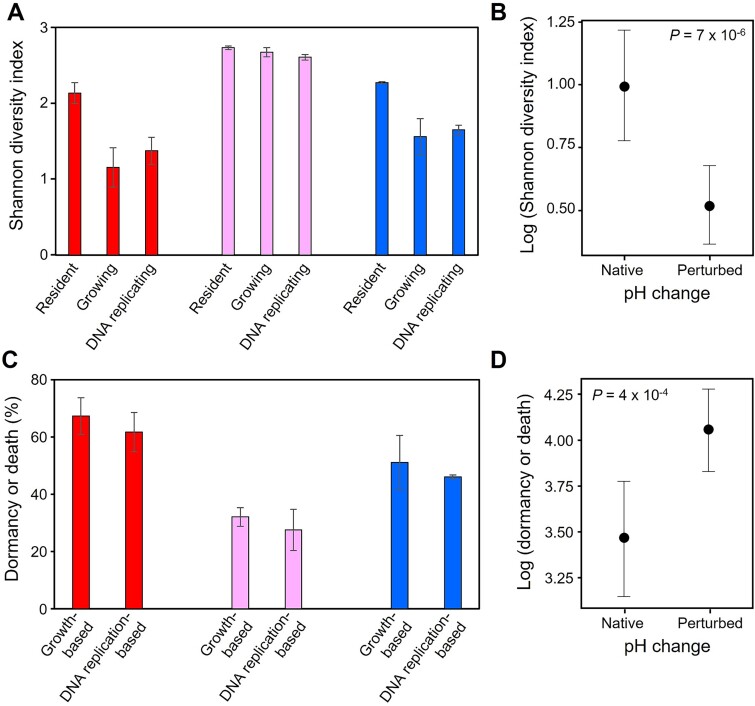
Shannon diversity index (A and B) and importance of dormancy (combined with death; C and D) in three incubated soils following environmental changes. In panel A, the Shannon diversity was estimated for the resident (DNA-based estimation), growing (DNA-based estimation), and DNA replicating (SIP-based estimation) AOA communities. In panel C, the proportion of dormancy or death was estimated using the growing (DNA-based estimation) or the DNA replicating (SIP-based estimation) AOA communities. Mean and standard error data are represented for observed (panels A and C) and predicted (panels B and D) values. The red, pink, and blue colors represent soil pH 4.5, 6.0, and 7.5, respectively.

The above results demonstrate that the ecological specialization state impacts the fitness of organisms following an environmental perturbation. The composition of the active communities was then investigated to assess their composition following environmental disturbances and identify how specific phylotypes contributed to any community changes. The community composition of active communities depended on the soil pH disturbance (Fig. 4), with community composition being more similar between DNA and SIP than RNA assessment. In the context of AOA taxonomy and pH niche breadth, most of the active specialists (and putative specialists) were affiliated to the acidophilic NT-Alpha and neutrophilic NP-Eta phylogenetic clusters, while active generalists belonged mainly to the neutrophilic NS-Delta, NS-Gamma-2.2, and NP-Gamma phylogenetic clusters (Fig. 4). In the native soil (pH 6.0), a similar Shannon diversity index in the resident and active communities suggests that most resident AOA phylotypes were growing and replicating (Fig. 5A). In contrast, the diversity of active AOA was lower for the pH-perturbed than the native pH soils (Fig. 5A and B).

As the pH disturbances often led to fewer active cells than in the native condition (Fig. 3A), the growth and activity of a restricted number of phylotypes was likely strongly selected following environmental change. Selection was based on ecological specialization, with the most abundant active phylotypes being generalists following an increase in soil pH and specialists (or putative specialists) following a decrease (Supplementary Table S2). Similarly, the activity of phylotypes with an opposite phenotypic state (e.g. generalists following a soil pH decrease) or having a non-optimal pH preference (e.g. acidophilic following a soil pH increase) decreased, suggesting that these phylotypes were outcompeted by favored phylotypes, through reduced activity or via the onset of dormancy or death. These findings highlight the importance of pH specialization and niche breadth state in maintaining microbial diversity and activity following environmental change.

Combined dormancy and death were also investigated as a mechanism of microbial adaptation. The percentage of inactive cells (including dormant and dead cells) in the pH-change experiment ranged between 32% and 67% (Fig. 5C) and was higher in pH-perturbed soils than in native conditions (Fig. 5D). There was no evidence of resuscitation from dormancy following the pH disturbance, as phylotypes active in pH-perturbed soils were already active in the native unperturbed soils (Supplementary Table S2).

### Change between specialist and generalist states over evolutionary time

**Figure 6:**
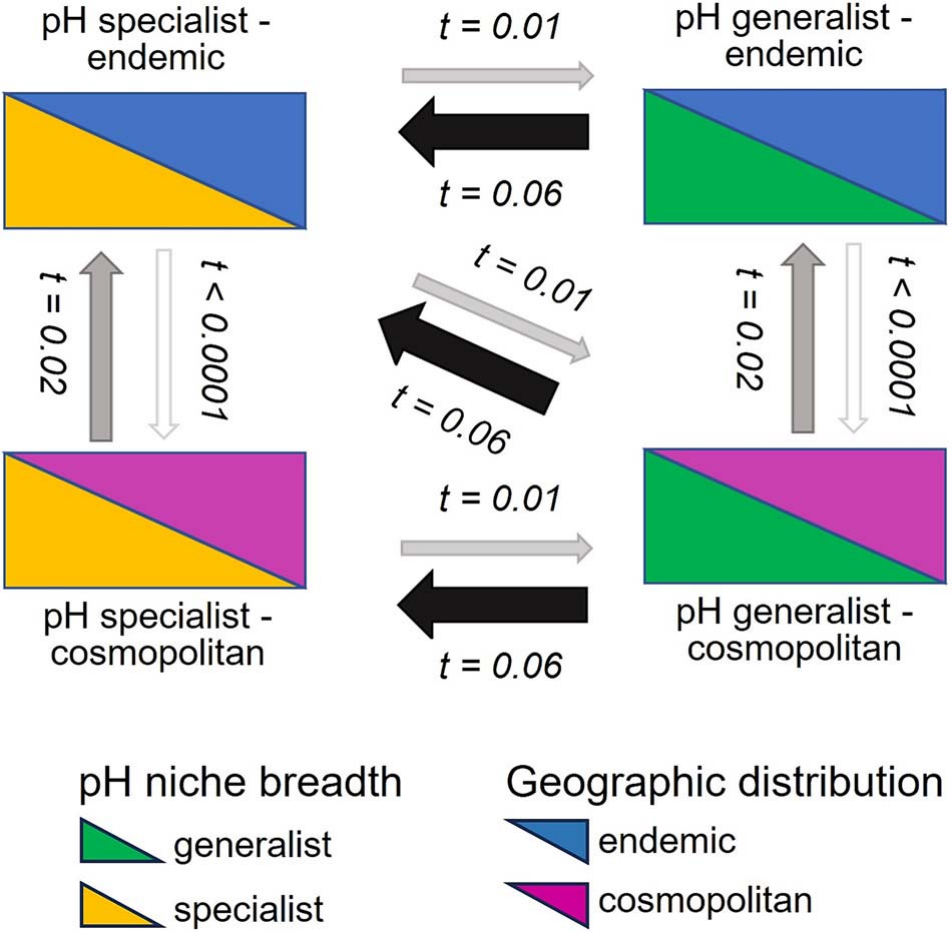
State model diversification of specialists and generalists over evolutionary time for the best binary-state model of niche specialization. Transition rates were estimated between the four-state systems, combining the pH specialist/pH generalist and the endemic/cosmopolitan phenotypes. Speciation and extinction rates were similar for each of the four states, but transition rates from the generalist to the specialist states were greater than the reverse. Increased transition rate (*t*) is represented by the widening and color darkening of the arrows.

The greater ecological fitness of generalists than specialists following a short-term environmental change and the greater adaptability of specialists to extremes may balance distributions over short time scales depending on the range of conditions over which environments fluctuate. However, distributions are also influenced by longer-term evolutionary transitions, speciation, and lineage extinction processes. To investigate these long-term mechanisms, the diversification rates of the specialists and generalists were analyzed over evolutionary time, and the transition rates between the two states were estimated. A substantial variation in diversification rates across lineages was detected, with homogeneous-rate models having weak support (Supplementary Table S3). In the best-performing model (model 6), the variation in diversification rate was decoupled from pH specialization, with lineages diversifying at the same rate regardless of their generalist or specialist status (Fig. 6). Therefore, speciation rates do not appear to be associated with faster adaptation of generalists to environmental changes, and increased ecological specialization does not appear to be associated with reduced speciation rates or increased extinction risk.

Despite similar diversification rates, the transition rate to a specialist state was six times greater than that of a generalist one (Fig. 6), suggesting that generalists thrive in various environments along their evolutionary history and then specialize in specific environments, becoming specialists. Indeed, ancestral state reconstruction indicates a higher likelihood of being a specialist than a generalist for all the phylogenetic nodes, suggesting that specialist lineages always seem to have evolved toward generalists (Supplementary Fig. S6). The preponderance of generalists in non-extreme, stable conditions and the high fitness of generalists following environmental change likely balance against the high evolutionary rate of gain in specialism and its benefit in extreme environments; these joint ecological and evolutionary forces contribute to the overall maintenance of microbial diversity.

The links between the ecological patterns of coexistence and the large-scale phylogenetic processes were further elucidated by analyzing the distribution of the specialist and generalist phylotypes across the AOA phylogeny (Fig. 1A). There was no evidence for phylogenetic signal or conservatism of the specialist/generalist traits (*K* statistic = 0.1; *P* value = .054). Such a low *K* statistic might imply phylogenetic overdispersion (sister species being less likely to share niche breadths than expected at random), showing that generalism and specialism are highly unlikely to be conserved through repeated speciation events, and suggesting that phylogeny does not dictate the specialization phenotype per se. The analysis of the phylogenetic relatedness in microbial communities distributed across a gradient of pH showed that phylotypes are also more closely related than expected by chance in soils with pH > 6 (as indicated by a significant negative ses.mpd; Fig. 1B). This suggests that soil pH favors selection of specific AOA communities, but not necessarily those with a specific niche breadth phenotype. The extent of evolutionary relatedness of AOA communities increases with pH level (relationship ses.mpd—pH: *R*^2^ = 0.28; *F* statistic = 17.2; *P* value = 1.5 × 10^−4^); however, linear models suggest that the presence of specialists does not strongly influence the change in phylogenetic structure of communities.

## Discussion

### Niche breadth specialization

This study supports the hypothesis that, for soil pH, archaeal generalists have a greater adaptation rate than archaeal specialists following environmental change, except in soils with extreme conditions where specialists are better competitors. Our evidence supporting this concept in the archaeal domain corroborates previous theoretical and experimental studies on bacteria, viruses, and eukaryotes [7, 17, 48–52], demonstrating the potential universality of this concept across the tree of life. Demonstrating this concept with soil pH change as an environmental disturbance strengthens evidence of its importance, and previous studies [7, 17, 49, 51, 52] have analyzed other factors, including resource limitation and oxygen fluctuations, and further testing could analyze more factors. In addition, niche specialization was recently shown to be multidimensional for soil prokaryotes, with generalization or specialization in one niche axis (e.g. pH) supporting similar niche trajectories in other niche dimensions (e.g. temperature, moisture, litter depth) [53].

The results are robust over several analytical scales, including the short- and long-term adaptation of archaeal communities, but apply to the active and not the resident microbial communities, which include active, growing, dormant, and dead cells (and free DNA). Whereas most microbial studies previously focused on resident communities, discriminating cells of different metabolic states is a major emerging challenge to understanding the mechanisms regulating their adaptation [54, 55]. The comparison of methodological activity estimations also highlighted the divergence of transcriptional activity from the growth and DNA replication estimations. Previous gene-, transcript-, and protein-based activity comparisons (e.g. [56]) reported transcript abundance as a strong predictor of activity during short-term incubation [57, 58], but this may not be the case in our more prolonged incubations, where substantial RNA may have been degraded [59]. In contrast, our growth or DNA replication estimates likely estimate microbial activity accurately over the incubation period, given their strong correlation with related ecosystem function (nitrification) [31]. Therefore, we suggest caution in using mRNA- [or ribosomal RNA (rRNA)-] based approaches in this context as likely reflecting potential, rather than actual, activity [60].

In this study, niche adaptation to environmental changes was demonstrated using AOA microorganisms. Despite absence of phylogenetic signal for niche specialization, some phylogenetic clusters indicated presence of active specialists (e.g. acidophilic NT-Alpha and neutrophilic NP-Eta) or active generalists (e.g. neutrophilic NS-Delta, NS-Gamma-2.2, and NP-Gamma). Microbial isolation (e.g. [61, 62]) and growth of some of these lineages in soil (e.g. [63]) were observed previously, but future studies should test the existence of specialists and generalists in these different phylogenetic lineages to confirm such a phenotypic niche breadth.

### Underlying genomic mechanisms of niche-specific adaptation

Whereas not the focus of the current study, several underlying genomic mechanisms of niche-specific adaptation can be suggested. Generalists’ adaptation to novel, non-extreme environments likely reflects their greater metabolic and physiological versatility and flexibility [64]. The latter can originate from numerous genes linked to diverse metabolic traits [16], probably related to environmental sensing, transport, and complex resource degradation functions [65], including extracellular proteins [66]. This genomic flexibility potentially results in generalists’ large genome size [67], or at least in high variability of their genome sizes [16]. Microbial genome size was previously reported to be larger in fluctuating than stable environments [68], and high physiological flexibility, allowing organisms to thrive over a broad range of environmental conditions, was recently advanced to explain bacteria thriving in marine sediments subjected to fluctuations in oxygen, light, nutrients, and redox state [17]. Novel gene acquisition, through lateral gene transfer, followed by intense gene duplication events, has been demonstrated as a critical source of molecular innovation driving thaumarchaeotal adaptation during their evolutionary history [19, 69] and may represent one key mechanism of evolutionary gain in generalism over long evolutionary time scales. We have looked for evidence of an overall larger genome size of generalists than specialists in AOA by analyzing many AOA genomes. Despite an increasing number of AOA genomes in databases, we concluded that the number currently available is still too limited to reach a reliable conclusion, and this hypothesis should be revisited when more genomes are available. In contrast to generalists, specialist genomes often encompass specific metabolisms with highly conserved functional genes [65], maximizing fitness in extreme conditions. Genomes of specialists have initially been advanced as being streamlined [70], preventing transitions back to generalism, which involves gene gain, and which is more complicated (see Fig. 6). This may be particularly true for specialists in extreme rather than non-extreme environments, as a negative correlation between niche breadth and genome size was shown in samples of high alpha-diversity (such as neutral or high pH soils) [16]. In addition to (potentially) lower genomic flexibility, specialists generally have a lower growth rate potential than generalists, based on genome prediction [16], facilitating their overgrowth by generalists in non-extreme conditions. The long-term dynamics of de novo evolutionary transitions between generalists and specialists are relevant for diversification dynamics which contribute to the regular maintenance of a range of available generalist and specialist genotypes and phenotypes across the landscape. These phylotypes can then sort themselves into communities following selection on standing genetic variation imposed by short-term dynamics of ecological transitions and patchily distributed environmental extremes.

### Associated ecological-evolutionary mechanisms of niche breadth specialization

High environmental persistence and survival by dormancy have been advanced as key mechanisms of microbial adaptation [6, 18, 71, 72], and maintenance of microbial diversity [12, 47, 73]. For example, increased dormancy was critical for resilience to warming press disturbance [74]. Here, dormancy (combined with death) was low compared to previous estimates in soil [47] and resuscitation from dormancy was not detectable following pH changes. While maintenance of soil at a stable pH for 70 years may have minimized the selective advantage of phylotypes able to enter dormancy when pH is not optimal, low dormancy and resuscitation rates may also result from specific thaumarchaeotal genomic traits. Indeed, a unique 16S rRNA copy in most Thaumarchaeota genomes [19] suggests low dormancy potential [75], based on their growth strategies. In addition, some genes commonly involved in microbial dormancy, such as the toxin-antitoxin systems (providing a mechanism for cell persistence to cope with various stress conditions) [76], are underrepresented in Thaumarchaeota [76]. Finally, a metabolic pathway currently recognized as critical and widespread for dormancy maintenance in many prokaryotes is the consumption of atmospheric hydrogen via hydrogen-oxidizing hydrogenases (groups 1 and 2) [77], whose genes are absent in Thaumarchaeota genomes [78, 79]. Nonetheless, future analyses could recover unrecognized defense and dormancy machineries in those organisms [76].

At the long-term evolutionary scale, the high transition rate from the generalist to the specialist state, confirming the existence of adaptive switching in generalists [10], may be explained by the “resource-spectrum engineering” theory [80], in which specialists can change the resource spectrum of the niche, leading to generalist state instability and a new specialist preferential state. This theory aligns well with the niche construction process where organisms adapt to and modify the external environment (e.g. the metabolite landscape), creating novel ecological niches and allowing more diversification [81]. For example, AOA acidify their nearby environment during nitrification, potentially limiting the growth of generalists in spatially isolated sites, e.g. soil pores. At the small spatial level relevant for microbes, frequent pH fluctuations occur in natural soils through cycles of acidification and neutralization linked to plant root exudates in the rhizosphere or intermittent ammonia oxidizer growth dependent on ammonia supply. These pH change cycles are likely associated with the long-term adaptation of generalists to overcome the unstable period, leading to the speciation of novel generalist phylotypes. Following the fluctuating period, old and new generalist lineages shift to the specialist state in the stable environment, explaining the high transition rate to this state. Therefore, this framework suggests that generalism is likely a transitional state in the microbial life history, with most phylotypes eventually becoming specialists. Furthermore, the very low phylogenetic signal in niche specialization implies that there is nothing unique to individual archaeal phylotypes or lineages in their capacities to become generalized or specialized—in fact, these transitions occur even more than expected by chance, given the branching pattern of the tree.

Our study, therefore, provides evidence of the importance of niche breadth for ecological and evolutionary adaptation following an environmental change in archaea, expanding the current theory to prokaryotes thriving in complex environments with a significant role in global biogeochemical cycling and adding evidence for the universality of currently accepted theory. This finding is critical to answering a wide range of crucial questions on microbial distribution and diversity in natural environments, which is essential in light of the biodiversity threat in challenged fluctuating ecosystems.

## Supplementary Material

20240917_msSG_SI_ISME_R3_wrae183

20240912_SG_SuppTables_wrae183

DataSources_SG_final_wrae183

## Data Availability

All statistical analyses are detailed in the data sources. Additional codes can be found at https://github.com/AigleAxel/amoA_MiSeq_sequencing/ and https://github.com/leonelhalsina/archaea_pH. The ammonia monooxygenase *amoA* AOA sequences for the incubated soil experiments can be found under the NCBI BioProject PRJNA1013403, whereas *amoA* sequences from the 47 soil samples (used for the database) can be retrieved as online supplementary material (Data S1) of a previously published paper (https://doi.org/10.1111/mec.13607). The updated *amoA* database containing the niche breadth index is included in the data source document associated to this manuscript.
