# Supplementary material for: Niche breadth specialization impacts ecological and evolutionary adaptation following environmental change
Source: ISME J. 2024 Sep 26;18(1):wrae183. doi: 10.1093/ismejo/wrae183 (PMC11630254; doi:10.1093/ismejo/wrae183)
Supplement: 20240917_msSG_SI_ISME_R3_wrae183 [file 20240917_mssg_si_isme_r3_wrae183.docx]

**Supplementary Information for "** **Niche breadth specialisation impacts ecological and evolutionary adaptation following environmental change"**

Cecile Gubry-Rangin, Axel Aigle, Leonel Herrera-Alsina, Lesley T Lancaster and James I Prosser

**Figures**

**
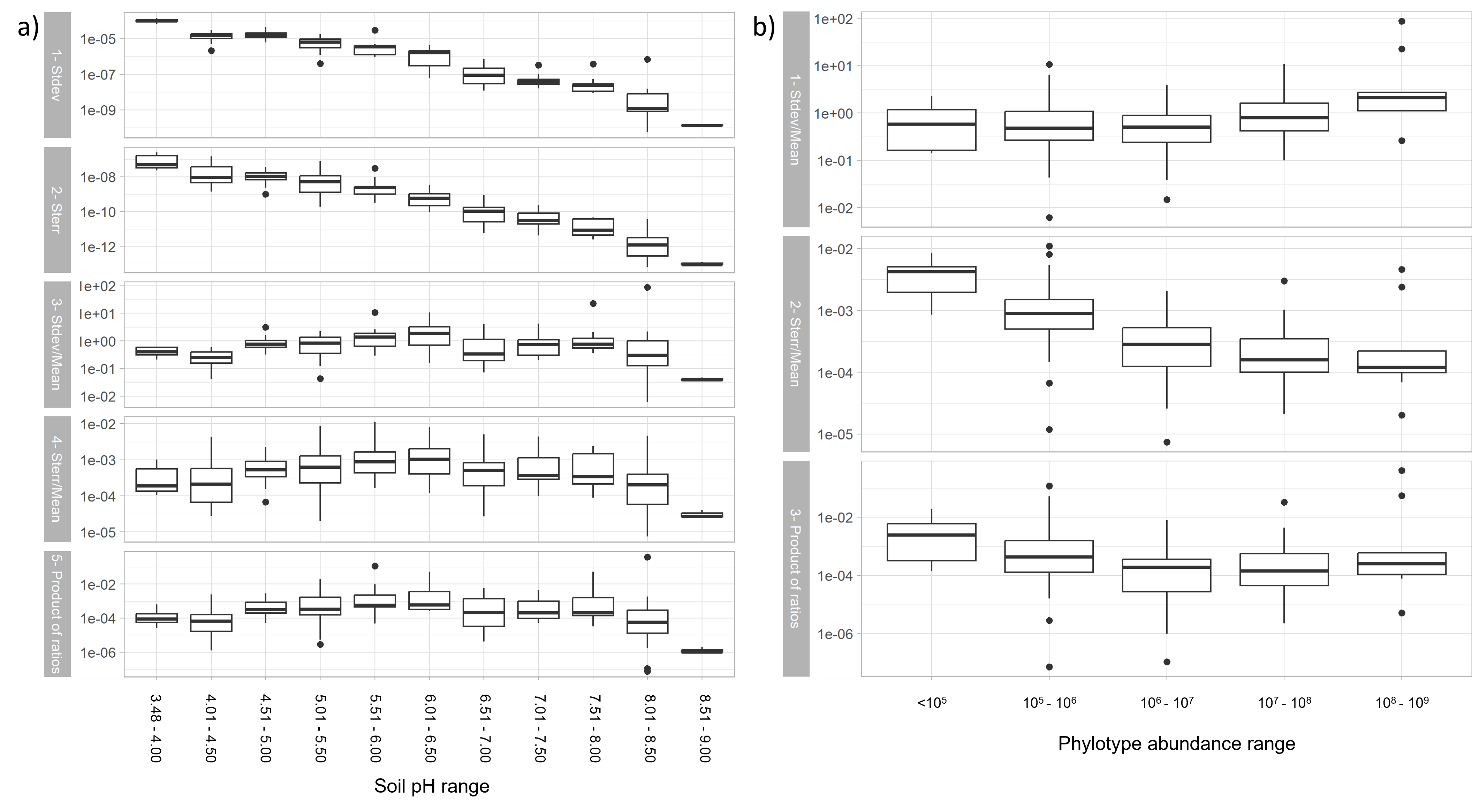
**

**Figure S1: Distribution of phylotype dispersion and niche breadth values in the database.** a) Distribution of phylotype dispersion values in the database based on the soil pH range. Several dispersion values were estimated for each phylotype based on its abundance in the different soils by considering the pH of each soil. The boxplots represent the distribution of those dispersion values. The mean corresponds to the average soil pH in which the phylotype is distributed and the standard deviation (Stdev = SD) and the standard error (Sterr = SE) represent the dispersion around the mean values for all sequences within each phylotype. As the standard deviation and standard error presented a skewed distribution toward the lower pH values across the pH range, they were divided by the pH mean. The product of these ratios was chosen as the niche breadth index as it was the least distorted across the pH range (see panel b). b) Distribution of phylotype dispersion values in the database based on the abundance of each phylotype. The mean, standard deviation and standard error definitions are those in panel a. Here, the ratios (Stdev/mean and Sterr/mean) presented a skewed distribution across the abundance range toward the higher or lower abundance values. Therefore, the product of these ratios was chosen as the niche breadth index as it was the least distorted across the abundance range. Statistical comparisons were performed using Kruskal Wallis ranking tests followed by pairwise Dunn tests.

**
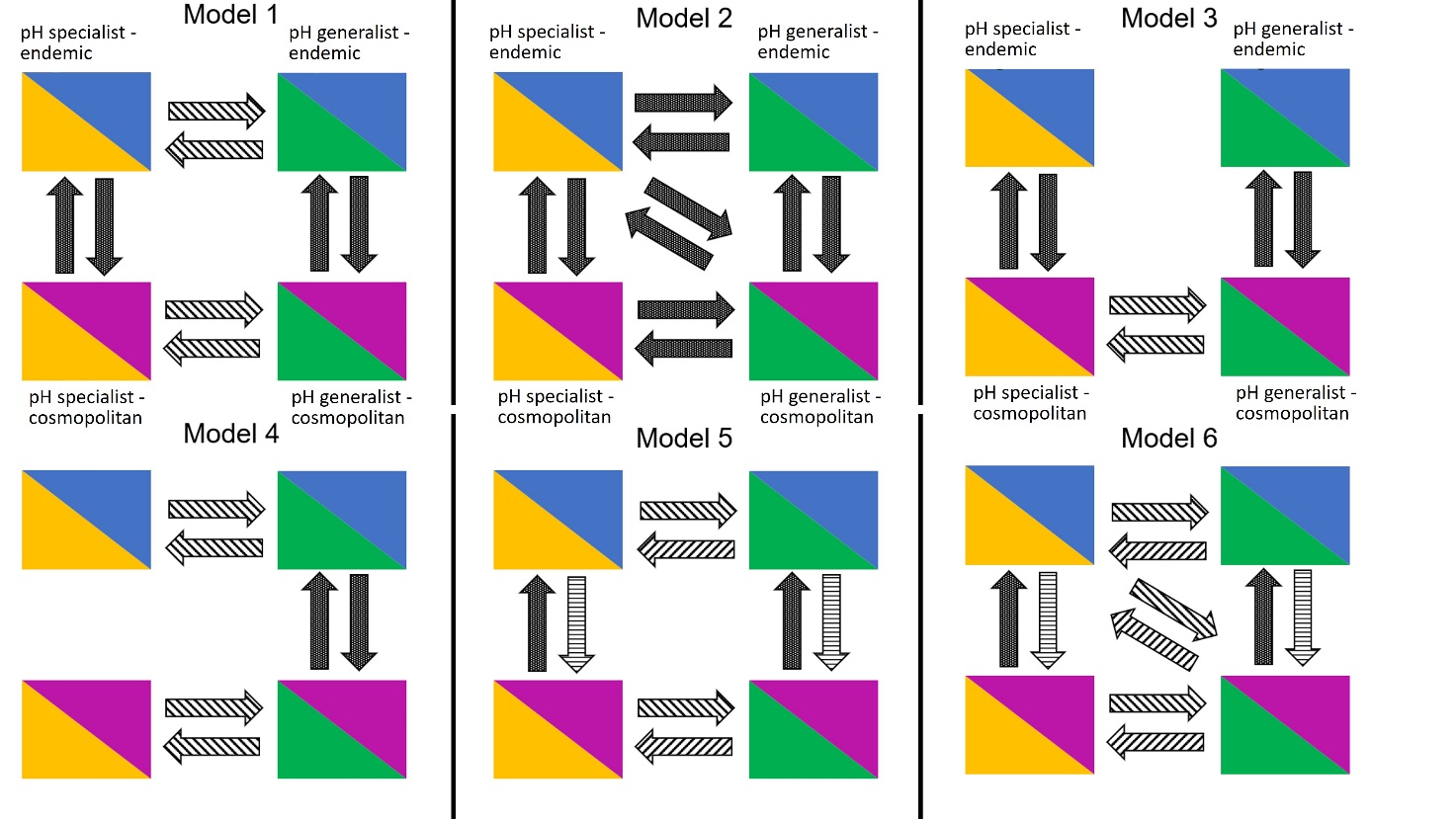
**

**Figure S2:** **Six models of pH preference and geographic range size evolution.** For each model, arrows indicate which transitions are allowed in the four-state system, and transitions which have the same rate are indicated by the same font colour. For example, in Model 2, there is only one rate for all allowed transitions whereas in Model 6 the same transitions are possible but there are four different rates. Models 3 and 4 assume that endemic-pH specialists are an evolutionary dead end and to reach this final state, it is necessary to have either range reduction (Model 3) or pH specialisation (Model 4). In Model 1, the rate of geographic range contraction/expansion is different from the rate of becoming generalist/specialist. In Model 5 the rates of pH specialisation differ from pH generalisation and range expansion takes place at a different rate than range contraction. We decided to omit the model allowing the transition from cosmopolitan-pH specialists toward endemic-pH generalist and the reverse as it would represent a drastic change of phenotype, likely impossible from a biological point of view.


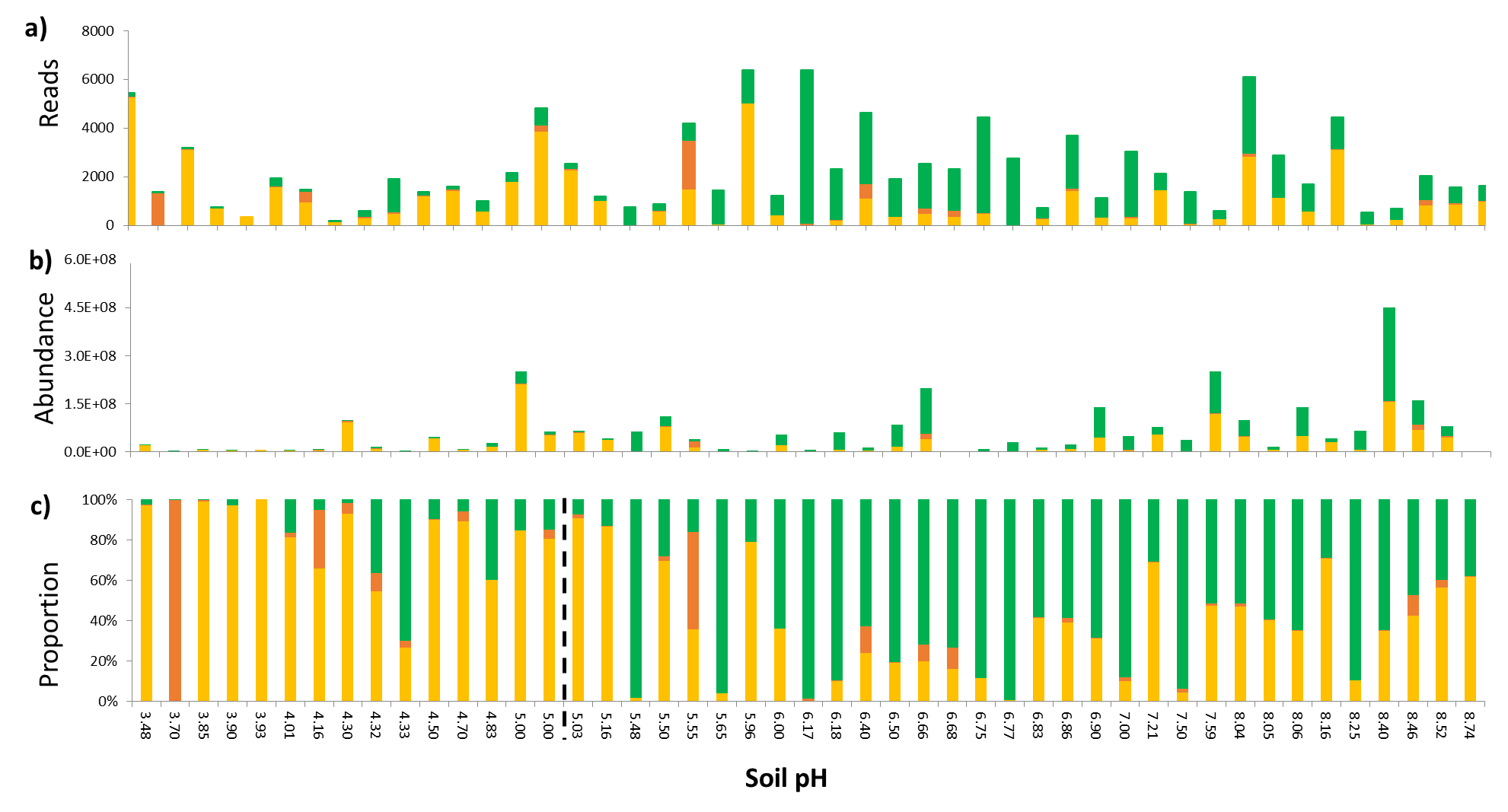


**Figure S3: Representation of (a) the number of sequencing reads, (b) the abundance of archaeal cells and (c) the proportion of archaeal cells in each of the 47 native soils.** The yellow, green and orange colours correspond to the specialist, generalist or putative-specialist categories. Soils are ranked by pH value. The lower pH soils contain a higher proportion of specialists than generalists and vice-versa, using the pH threshold represented by the dotted line for statistical analysis.

**
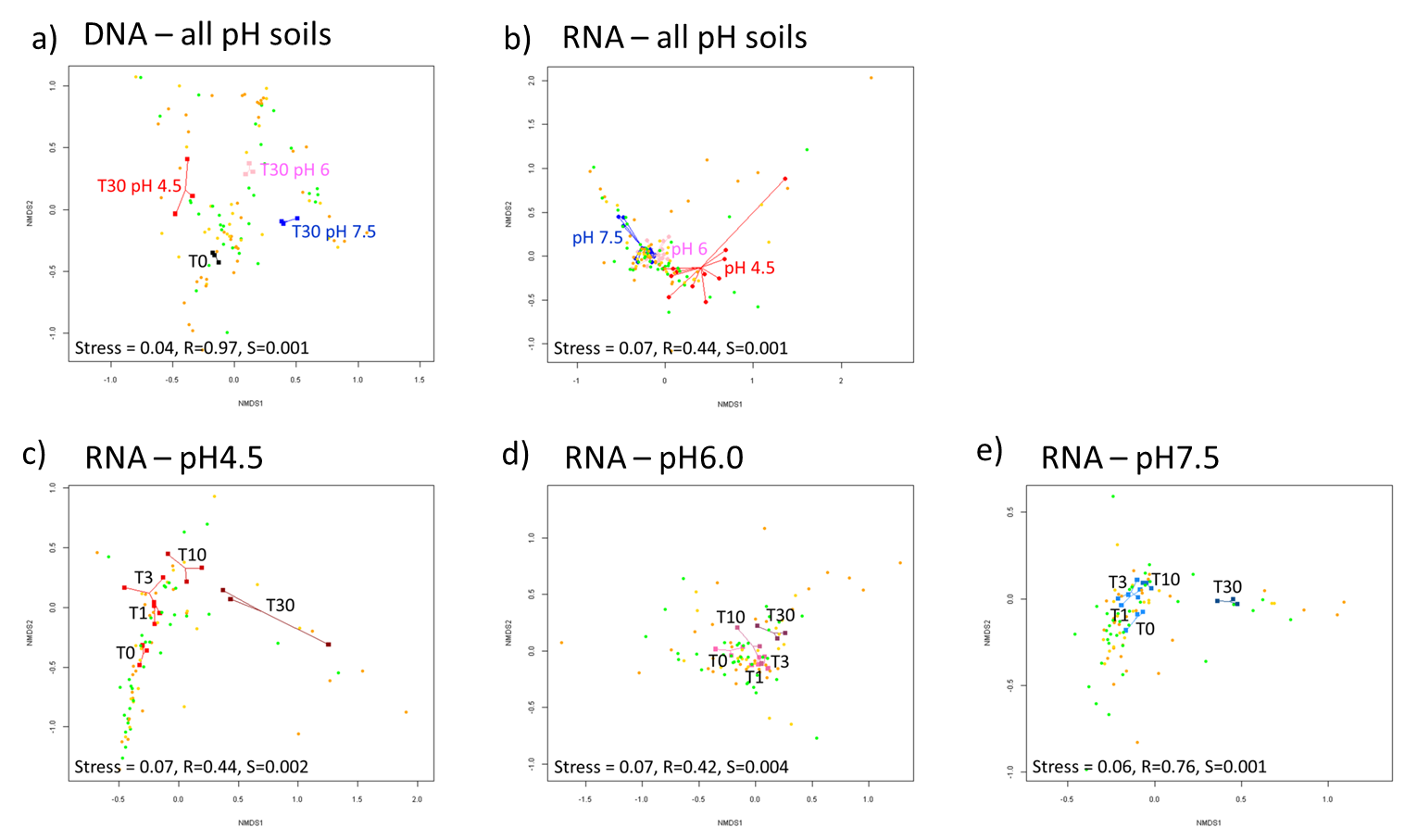
Figure S4:** **Community analysis of the thaumarchaeotal communities present following environmental changes.** The similarity between different resident communities is represented by Bray-Curtis dissimilarity nonmetric multidimensional scaling (NMDS) plots. (a) the *amoA* gene community composition at time 0 (T0) and after incubation for 30 days either maintained at pH 6 (T30 pH 6) or subjected to a pH change from pH 6 to pH 4.5 (T30 pH 4.5) and pH 7.5 (T30 pH 7.5). (b) the *amoA* transcript community composition after incubation for 30 days either maintained at pH 6 (pH 6) or subjected to a pH change from pH 6 to pH 4.5 (pH 4.5) or to pH 7.5 (pH 7.5) (c, d, e) the *amoA* transcript community composition time series (time 0, 1, 3, 10 and 30 days) maintained at pH 6 (d) or subjected to a pH change from pH 6 to pH 4.5 (pH 4.5) (c) or to pH 7.5 (pH 7.5) (e). Stress values are presented alongside each NMDS plot, differences between groups were tested by ANOSIM (ANOSIM statistic R), and the associated significance (S) values are also presented alongside each NMDS plot (number of permutations = 999). Samples are represented by squares and coloured according to their pH with red (pH 4.5), pink (pH 6.0) and blue (pH 7.5). Each phylotype is represented by a circle and coloured according to its niche specialisation category (specialist (yellow), generalist (green) and putative specialist (orange)).


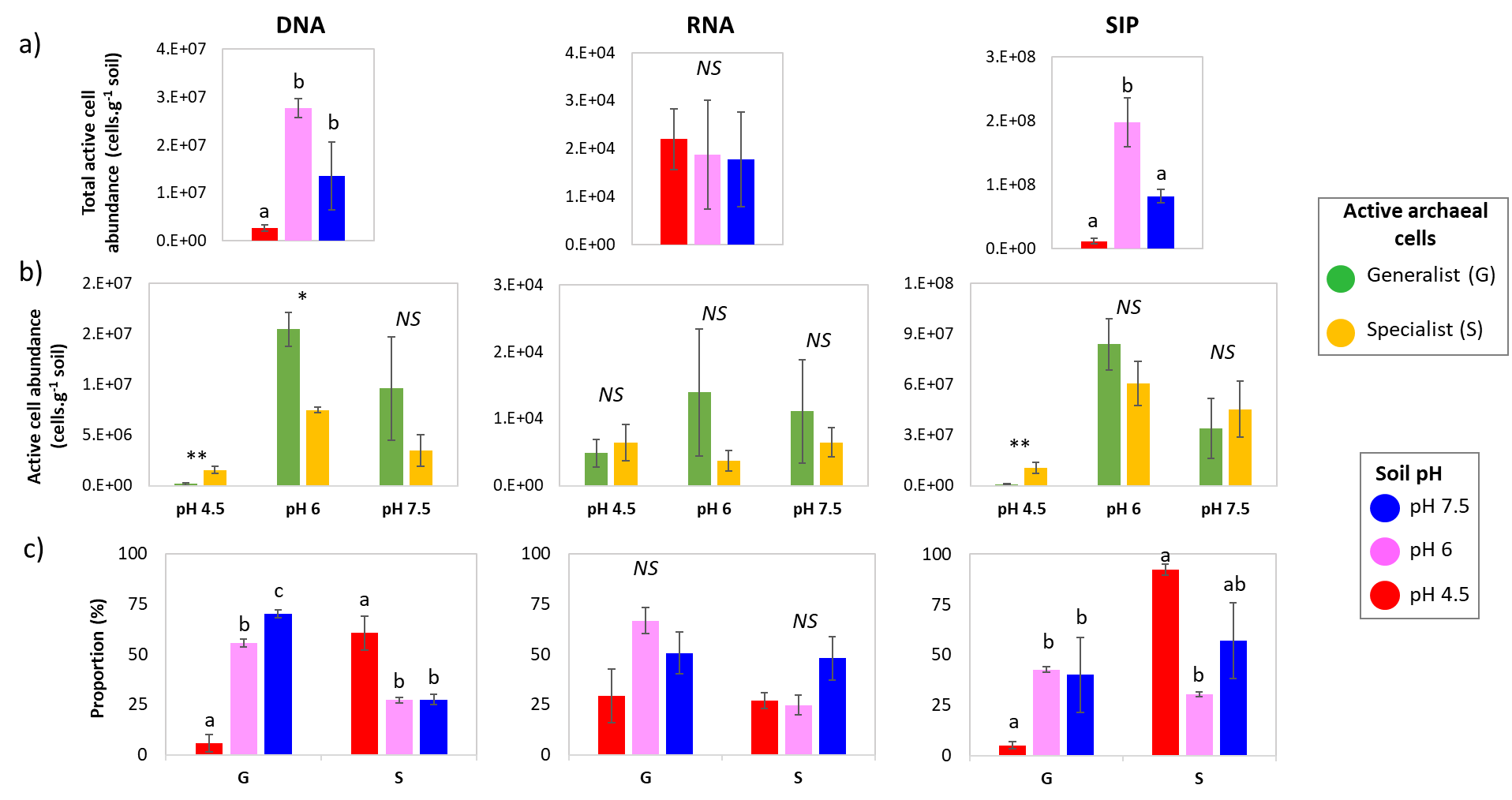


**Figure S5:** **Abundance and proportion of active specialist, generalist and putative specialist active archaea and total active AOA cells** **in three incubated pH soils following environmental changes using three activity estimates.** The abundance (top panels) and proportion (bottom panels) of specialist, generalist or putative-specialist AOA cells were estimated within soil microcosms either maintained at their native soil pH (pH 6) or subjected to a pH change (from pH 6 to pH 4.5 or to pH 7.5) for 30 days of incubation at 25°C. The activity of AOA cells was estimated by their growth (DNA), their transcriptional activity (RNA) and their DNA replication (SIP) after incubation for 30 days. Data are presented as the mean and standard error. For each figure, ANOVA and Tukey HSD (or Kruskall-Wallis) tests were performed to assess significance of differences between groups within each treatment (e.g. per pH in panel b and per phenotype in panel c). Different letters denote significant differences (*p*<0.05) between groups, while NS indicates non-significant differences between groups.

**
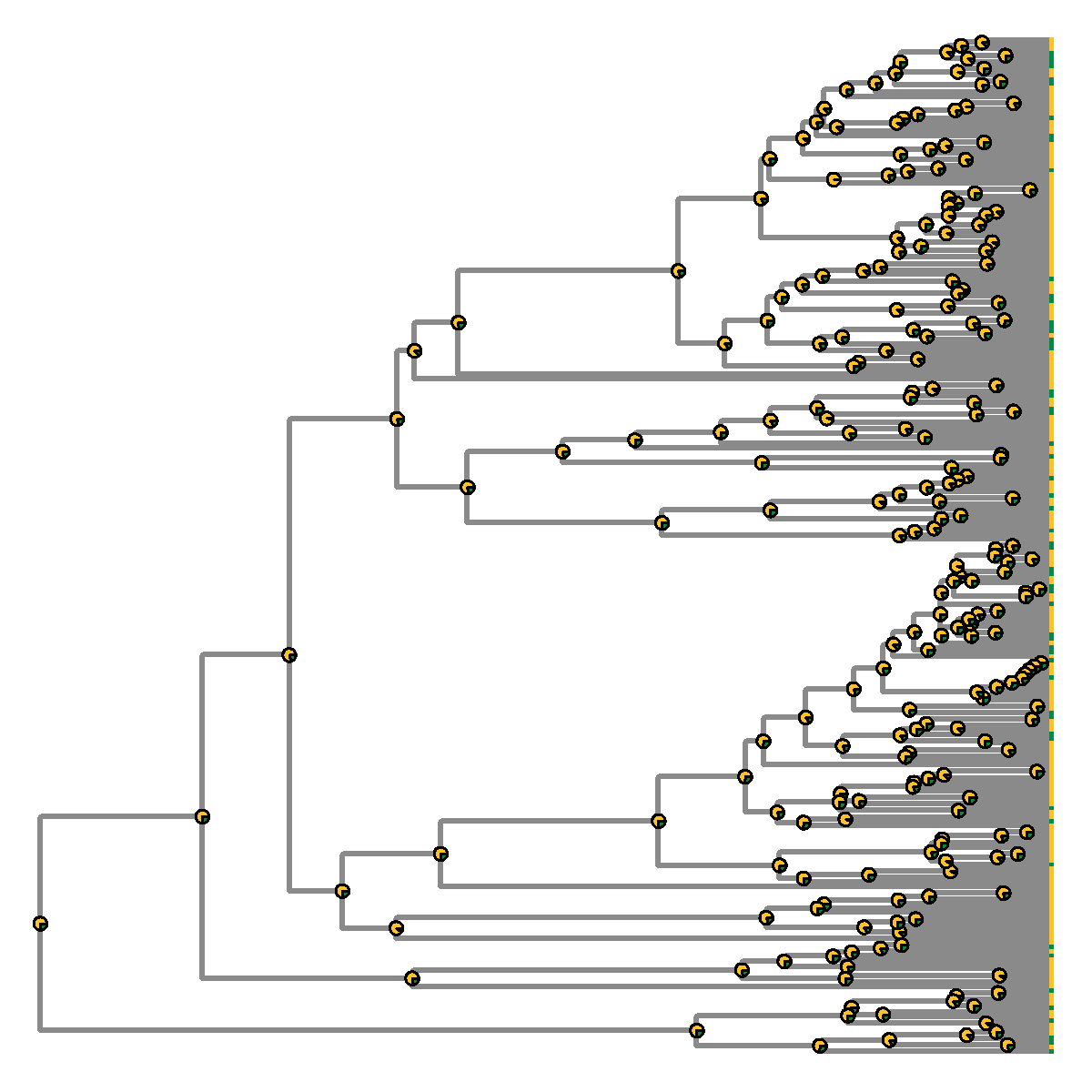
**

**Figure S6: Ancestral state reconstruction of niche specialisation status across the AOA phylogenetic tree.** The likelihood of each phenotype state is represented by the proportion of the pie chart on each node. Yellow and green correspond to specialist and generalist, respectively.
